# Supplementary material for: Pinning and hysteresis in the field dependent diameter evolution of skyrmions in Pt/Co/Ir superlattice stacks
Source: Sci Rep. 2017 Nov 9;7:15125. doi: 10.1038/s41598-017-15262-3 (PMC5680206; doi:10.1038/s41598-017-15262-3)
Supplement: Supplementary file 1 — Supplementary Information [file 41598_2017_15262_MOESM1_ESM.pdf]

SUPPLEMENTARY INFORMATION

Pinning and hysteresis in the field dependent diameter evolution of skyrmions in Pt/Co/Ir superlattice stacks

K. Zeissler[1*], M. Mruczkiewicz[2], S. Finizio[3], J. Raabe[3], P. M. Shepley[1], A.V. Sadovnikov[4,5], S.A. Nikitov[4,5], K. Fallon[6], S. McFadzean[6], S. McVitie[6], T. A. Moore[1], G. Burnell[1], and C. H. Marrows[1]

[1]*School of Physics and Astronomy, University of Leeds, Leeds LS2 9JT, United Kingdom*

[2]*Institute of Electrical Engineering, Slovak Academy of Sciences, Dúbravská cesta 9, 841 04 Bratislava, Slovak Republic*

[3]*Swiss Light Source, Paul Scherrer Institute, 5232 Villigen, Switzerland*

[4]*Laboratory "Metamaterials", Saratov State University, Saratov 410012, Russia*

[5] *Kotel'nikov Institute of Radioengineering and Electronics, Russian Academy of Sciences, Moscow 125009, Russia*

[6] *School of Physics and Astronomy, University of Glasgow, G12 8QQ, United Kingdom*

*Correspondence to k.zeissler@leeds.ac.uk

EXCHANGE STIFFNESS MEASUREMENTS

The exchange stiffness was extracted from the temperature dependence of the saturation magnetisation which can be described by the Bloch law sufficiently far below the Curie temperature. The Bloch law is given by [1, 2]

$$M(T)/M_S = 1 - C\left(\frac{k_B T 4 S^2}{A a}\right)^{3/2}, \quad \text{(S1)}$$

where C = 0.0294 in case of the fcc lattice that is the case here, S = 1, k_B is the Boltzmann constant, T is the temperature, and a = 0.355 nm is the lattice constant.

DMI MEASUREMENTS

Two methods were used to extract the DMI strength: asymmetric bubble expansion and Brillouin light scattering (BLS). The bubble expansion gives a localized value of D at pinning sites whereas BLS returns the average measured over a larger area and hence is less susceptible to local pinning potentials.

In the asymmetric bubble expansion method, a bubble was nucleated in a single trilayer [3, 4]. An out-of-plane magnetic field was pulsed and domain wall creep velocity was extracted from the visible bubble expansion. The domain wall velocity was measured for different static in-plane fields which act with or against the DMI field at the domain wall on either side of the bubble, modifying the wall energy (see figure Supplementary 1 (a)). The minima in the velocity versus in-plane field shows the point where the in plane field cancels the effective in plane field induced at the wall by the DMI, H_{DMI}, and can be used to extract the DMI strength D using the formula

$$D = \mu_0 H_{\mathrm{DMI}} M_{\mathrm{S}}\, \Delta, \quad \text{(S2)}$$

where M_S is the saturation magnetisation and Δ is the domain wall width and is given by $\sqrt{A/K_{eff}}$, in which A is the exchange stiffness and K_{eff} is the effective perpendicular anisotropy constant. Two trilayer films were measured (Ta(4.8 nm)/Pt(6.2 nm)/Co(0.5 nm)/Ir(1.5 nm) and Ta(3.7nm)/Pt(4.5 nm)/Co(1.0 nm)/Ir(3.0 nm)). D was measured to be 0.6$\pm$0.1 mJ/m^2 for the 0.5 nm cobalt layer and

0.3±0.1 mJ/m2 for the 1.0 nm layer. In the Brillouin light scattering method [5, 6], the DMI strength is extracted by measuring an asymmetry in the Stokes and anti-Stokes frequencies of light that has been inelastically scattered from propagating spin waves. In a sample with notable DMI strength, spin waves of a given wavelength propagating in opposite directions have different energies. This behaviour is known as propagation nonreciprocity and occurs when the sample is magnetised in-plane and the spin wave vector is perpendicular to the magnetisation, the Damon-Eshbach geometry. The frequency shifts of the inelastically scattered light with respect to the incident laser beam frequency is directly proportional to the DMI strength

$$\Delta f = f_S - f_{AS} = \frac{2\gamma}{\pi M_S} D k_{SW}, \tag{S3}$$

where k_{SW} is the magnon wavevector, f_S is the Stokes frequency, f_{AS} is the anti-Stokes frequency, and γ is the gyromagnetic ratio. DMI was calculated using $D=(\Delta f\ \pi M_S t)/(2\gamma k)$ where γ=190 GHz/T is the gyromagnetic ratio, and t is the thickness. The saturation magnetisation M_S was measured to be 1.1 MA/m for this sample. The Stokes and anti-Stokes spectra measured for k=7.1 can be seen in supplementary figure 1 (b). Supplementary figure 1 (c) shows the frequency shift for different wave vectors at positive and negative field. The average D was found to be 0.93±0.07 mJ/m^2 for the Ta(3.7nm)/Pt(4.5 nm)/Co(1.0 nm)/Ir(3.0 nm)) trilayer. BLS returns an average value over a µm length scale and is therefore less sensitive to local defects. Hence when treating polycrystalline samples we can regard D obtained by asymmetric bubble expansion as a lower limit and results obtained using BLS as an average value [7, 8]. Bubble expansion requires a reproducible nucleation point. In most cases this takes form of a large growth defect at the surface (see supplementary figure 1 (a) inset) and hence the surrounding area which is measured by the bubble expansion technique, is unlikely to be a representation of the average sample. Asymmetric bubble expansion is a technique very susceptible to local defects [7, 8].

EFFECTIVE THICKNESS MICROMAGNETIC SIMULATION

Supplementary figure 1 (d) shows the simulated defect free skyrmion diameter as function of magnetization. The N=10 repeat stack is compared to the effective thickness simulation where only one thick magnetic layer is considered. This shows that an effective thickness simulation is a good approximation of the multilayer system.

GRAIN SIZE OF POLYCRYSTALINE Pt/Co/Ir

Bright field transmission electron microscopy images where acquired on a representative trilayer Pt/Co/Ir polycrystalline sample. Ten images where taken at x50k and ten images where taken x80k magnification (see supplementary figure 1 (e) for an image taken at x50k). In total 20 different locations on the sample were investigated. Supplementary figure 1 (f) shows the percentage area of the different grain sizes observed, whilst the inset shows the frequency of the observed grains sizes. Whilst around 2/3 of the sample area is covered by grains less than 7 nm diameter, a second peak in the distribution was observed around 10 nm with 1/3 of the sample area being covered with these larger grains. This bi-modal distribution is not atypical for sputtered films in this thickness range. According to Kim et al. [9] magnetic skyrmions get pinned most strongly by grains of comparable size. In our structures the skyrmion has a diameter of 130 nm-270 nm and hence modelling the disorder with the average large grain size of 10 nm is reasonable for the observed distribution of grain sizes.

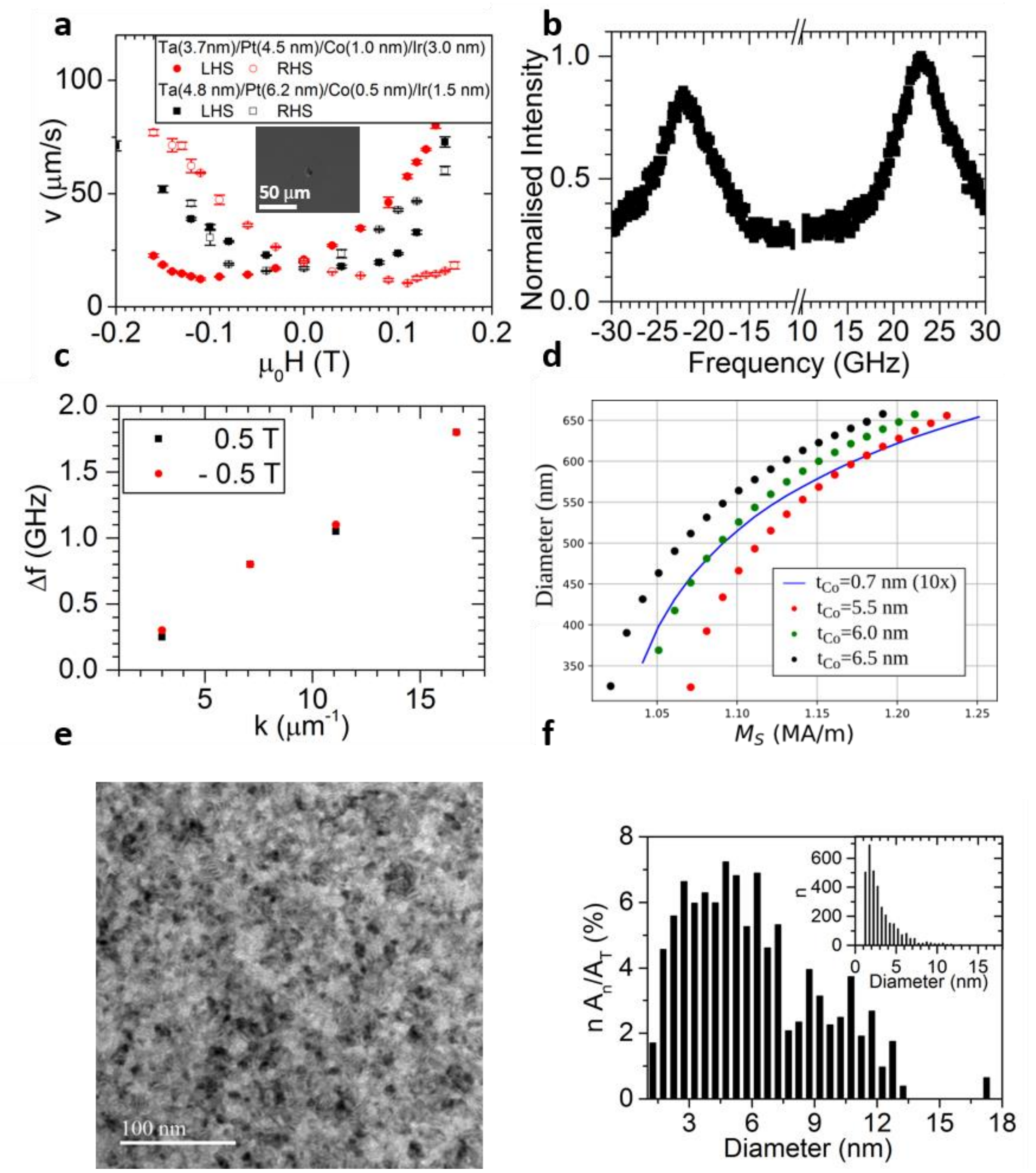

Supplementary Figure 1: (a) Domain wall creep velocity dependence on an in-plane field. The minima shows then DMI field. The inset shows an optical microscope image of a micrometer sized growth defect which acts as a nucleation site. (b) BLS Stokes and anti-Stokes peak at 0.5 T in plane field. The maximum peak frequency shift was measured to be Δf=0.8 GHz with an incident angle of 17.5° corresponding to a spin wave vector, k = 7.1 μm^{-1}. (c) Frequency shift versus wave vector in the BLS measurement of the 1.0 nm thick Co layer. (d) Skyrmion diameter as function of magnetization simulated for a multilayer stack of 10 Co layers in comparison to simulations of a single Co layers with a similar effective thicknesses. This shows that an effective thickness simulation is a good approximation of the multilayer system. (e) Bright field transmission electron microscopy image of a representative polycrystalline trilayer Pt/Co/Ir sample. (f) The percentage area coverage as a function of grain diameter, by grains of different sizes, nA_n/A_T where n is the number of grains with the same diameter, A_n is the area

1. Aharoni A. *Introduction to Theory of Ferromagnetism*. Oxford University Press (2000).

2. Chikazumi S. *Physics of Ferromagnetism*. Oxford University Press (1997).

3. Hrabec A*, et al.* Measuring and tailoring the Dzyaloshinskii-Moriya interaction in perpendicularly magnetized thin films. *Phys Rev B* **90**, 020402 (2014).

4. Je SG, Kim DH, Yoo SC, Min BC, Lee KJ, Choe SB. Asymmetric magnetic domain-wall motion by the Dzyaloshinskii-Moriya interaction. *Phys Rev B* **88**, 214401 (2013).

5. Kostylev M. Interface boundary conditions for dynamic magnetization and spin wave dynamics in a ferromagnetic layer with the interface Dzyaloshinskii-Moriya interaction. *J Appl Phys* **115**, (2014).

6. Moon JH*, et al.* Spin-wave propagation in the presence of interfacial Dzyaloshinskii-Moriya interaction. *Phys Rev B* **88**, 184404 (2013).

7. Gross I*, et al.* Direct measurement of interfacial Dzyaloshinskii-Moriya interaction in X vertical bar CoFeB vertical bar MgO heterostructures with a scanning NV magnetometer (X=Ta, TaN, and W). *Phys Rev B* **94**, 064413 (2016).

8. Soucaille R*, et al.* Probing the Dzyaloshinskii-Moriya interaction in CoFeB ultrathin films using domain wall creep and Brillouin light spectroscopy. *Phys Rev B* **94**, 104431 (2016).

9. Kim JV, Yoo MW. Current-driven skyrmion dynamics in disordered films. *Appl Phys Lett* **110**, 132404 (2017).
